# Young, Black/African American, and Latino communities are left behind despite legislative efforts in California to reduce HIV/STI disparities

**DOI:** 10.3389/frph.2023.1179334

**Published:** 2023-09-14

**Authors:** Tommi L. Gaines, Dan Werb, Orlando Harris

**Affiliations:** ^1^ Department of Medicine, University of California San Diego, La Jolla, CA, United States; ^2^ Centre on Drug Policy Evaluation, MAP Centre on Urban Health Solutions, St. Michael’s Hospital, Toronto, ON, Canada; ^3^ Department of Community Health Systems, School of Nursing, University of California, San Francisco, San Francisco, CA, United States

**Keywords:** HIV, sexually transmitted infections, legislation, California, health disparities, policy, youth, racial and sexual minorities

## Abstract

**Objectives:**

Sexually transmitted infections (STI) have been on the rise in the United States with racial/ethnic minority groups, gay and bisexual men, and youth experiencing the highest STI and HIV infection rates. In 2022, California became the first state in the nation to pass legislation, Senate Bill 306 (SB 306), requiring health care plans to cover the costs of home test kits for STIs, including HIV. This study examines provisions within SB 306 and its potential to reduce STI and HIV disparities among key demographic groups and geographic regions within California.

**Study design:**

Ecological cross-sectional study involving 58 California counties.

**Methods:**

Descriptive statistics and choropleth maps compared HIV/STI prevalence rates, uninsured rates, demographic composition, and healthcare provider coverage across California counties. Three geographically weighted Poisson regression analyses were conducted to separately examine the association between proportion of uninsured and HIV, gonorrhea, and chlamydia prevalence rates.

**Results:**

HIV/STI rates were significantly and positively associated with the proportion of uninsured residents in Central and Southern California counties. These counties had a higher proportion of demographic groups vulnerable to HIV/STI including a large Latino, Black/African American, and younger (age 15–24) population but had a lower rate of healthcare providers with prescription authority for home testing kits, which is a requirement under SB 306.

**Conclusions:**

Cutting-edge solutions are needed to stem the rising tide of new STI and HIV infections. While SB 306 is novel and innovative in intent, its coverage gaps will increase disparities and inequities among historically underserved populations.

## Introduction

On January 1, 2022, California became the first state in the United States (U.S.) to require healthcare plans and insurers to cover the costs of home testing kits for HIV and other sexually transmitted infections (STIs). Senate Bill 306, passed by the California legislature and known as the STI Coverage and Care Act, aims to reduce barriers to STI services, including STI and HIV testing, and expand access to other services at-home and in the community for patients and their partners (1).

This legislation came after years during which the number of STIs alarmingly increased across California. Rates of chlamydia, gonorrhea, and syphilis rose 40% from 2013 to 2019 with California having the highest number of infections in the nation (2, 3). The Centers for Disease Control and Prevention estimates that 1 in 5 people in the United States have an STI, which costs the California healthcare system an estimated $1 billion annually (4, 5). Young people and certain racial and ethnic groups are particularly impacted by a substantial burden of HIV and STIs (3, 6, 7). Prior to Senate Bill 306, efforts were already underway to curb the HIV epidemic in specific regions of California. In 2019, the U.S. Department of Health and Human Services launched a 10-year initiative, Ending the HIV Epidemic (EHE), with a focus on reducing new HIV infections by scaling up key HIV prevention strategies including HIV testing (8). The initiative focuses on 48 U.S. counties with a high burden of HIV defined as EHE jurisdictions; these include eight counties in California that have been targeted as key sites for scale-up of HIV prevention and treatment interventions.

When Senate Bill 306 was signed into law, excitement among community-based organizations and public health agencies was high since its intent was to make HIV and STI testing affordable and accessible for all Californians (9, 10). A comprehensive at-home STI self-testing kit that is commercially available costs on average $289 out of pocket, which is cost prohibitive, especially for communities that need it the most. Multiple studies have found higher screening uptake occurs when individuals conduct at-home HIV/STI self-testing compared to clinic-based testing (11). For many communities, especially those who experience higher rates of stigma and discrimination or have higher levels of medical distrust, home-based testing is preferred due to its convenience, ease of use, and privacy (12–15).

Senate Bill 306 defines home test kits as a product that allows individuals to self-collect specimens for STI and HIV in a remote location outside of a clinical setting. The home test kits must be approved or cleared by the U.S. Food and Drug Administration (FDA) for home use and approved for waiver under the Clinical Laboratory Improvement Amendments of 1988 (CLIA), where CLIA-waived tests are required to be simple to use and have a low risk of producing incorrect results.

Other important provisions within the law that must be met before tests will be covered by a health insurance plan include the requirement that home test kits be deemed medically necessary or appropriate, that home test kits are ordered by an in-network clinician, or that prescriptions are furnished through a standing order for patient use based on clinical guidelines. However, the latter requirement of a standing order for accessing comprehensive STI home test kits is different when compared to the FDA approved oral HIV home test kits, which are commercially available over the counter without a prescription or a standing order from a medical provider (16).

There have been other public health initiatives that sought to reduce disease incidence but have unintentionally exacerbated health disparities (17–20). We propose that key provisions within Senate Bill 306 have the potential to serve as a barrier to accessing home test kits and could lessen the public health impact of this legislation. Therefore, the aim of this study is to examine the anticipated impact of Senate Bill 306 on reducing HIV and STI disparities among key demographic groups (i.e., young people, racial/ethnic minorities, and uninsured) and across geographic regions in California with an emphasis on the eight EHE jurisdictions with a high HIV burden.

## Methods

The present study combined publicly available data from multiple sources that included HIV/STI prevalence rates, census demographics, and healthcare providers. The unit of analysis was California counties (*n* = 58).

### HIV/STI rates

The prevalence rates for chlamydia, gonorrhea, and HIV, per 100,000 persons by California counties were obtained from the California Department of Public Health STD surveillance data and AIDSVu—an interactive map of the HIV epidemic across national, state and county levels (21, 22).

### Demographics

Population demographic variables were obtained from the U.S. Census Bureau 2020 American Community Survey 5-year estimates (23). The primary independent variable of interest was the proportion of uninsured residents at the county level. Additional demographic summaries were extracted at the county level and included the proportion of Latino, Black/African American, and young individuals aged 15–24 years.

### Healthcare providers

The number of healthcare providers per 100,000 persons by California counties were obtained from the Area Health Resources Files available through the Health Resources and Services Administration (24). Health professionals included medical doctors, nurse practitioners, and physician assistants as these are the healthcare professionals with prescription authority for STI home testing kits.

### California EHE jurisdictions

Our analysis includes a focus on eight counties in California classified as EHE jurisdictions by the U.S. Department of Health and Human Services (8). In Northern California, these counties include Almeda County, Sacramento County, and San Francisco County. In Southern California, these counties include Los Angeles County, Riverside County, San Bernardino County, and San Diego County.

### Analysis

Descriptive statistics were generated to summarize HIV/STI prevalence rates and the demographic composition of California counties with a focus on the EHE jurisdictions. Choropleth maps were generated to visualize the spatial pattern of the demographic composition and healthcare providers at the county level. Mapped data was partitioned into terciles allowing for an equal number of data values per class. To account for spatially varying relationships, we applied a geographically weighted Poisson regression (GWPR) (25) and examined the bivariate association between each outcome variable (HIV, chlamydia, and gonorrhea) and the primary independent variable, proportion uninsured. Under GWPR, local regression coefficients and standard errors were calculated. We derived local critical values using pseudo *t*-statistics by dividing each local regression coefficient by the corresponding local standard error. The resulting pseudo t-statistics were mapped to visualize spatially significant associations at the county level (26). Data were analyzed using STATA 16.0 (STATA, College Station, TX) and ArcGIS Pro 2.9 (ESRI, Redlands, CA).

### Ethics approval

Institutional Review Board approval was not required since all data were aggregate summaries at the county level and did not meet the definition of human subject research.

## Results

Table 1 displays the descriptive statistics for the study variables. Across the 58 counties, prevalence rates for HIV, gonorrhea, and chlamydia were 226.2, 139, and 339.9 per 100,000 people, respectively. Approximately 7% of the California population was uninsured.

**Table 1:** Descriptive summary of HIV/STI prevalence and demographic composition in California counties (*n* = 58).

	Mean (SD)	Range
HIV/STI prevalence (2020)		
HIV prevalence per 100,000	226.2 (210.9)	0–1,515
Gonorrhea prevalence per 100,000	139.0 (82.6)	0–473
Chlamydia prevalence per 100,000	339.9 (148.2)	13–660
Population demographics		
% Black/African American	2.8 (2.8)	0–13.2
% Latino	31.6 (18.4)	5.8–85.2
% Age 15–24 years	12.6 (3.2)	6.6–23.9
% uninsured	6.7 (2.0)	3.2–12.6
No. healthcare providers per 100,000	350.3 (176.8)	89.4–1,154.3

**Figure 1:**
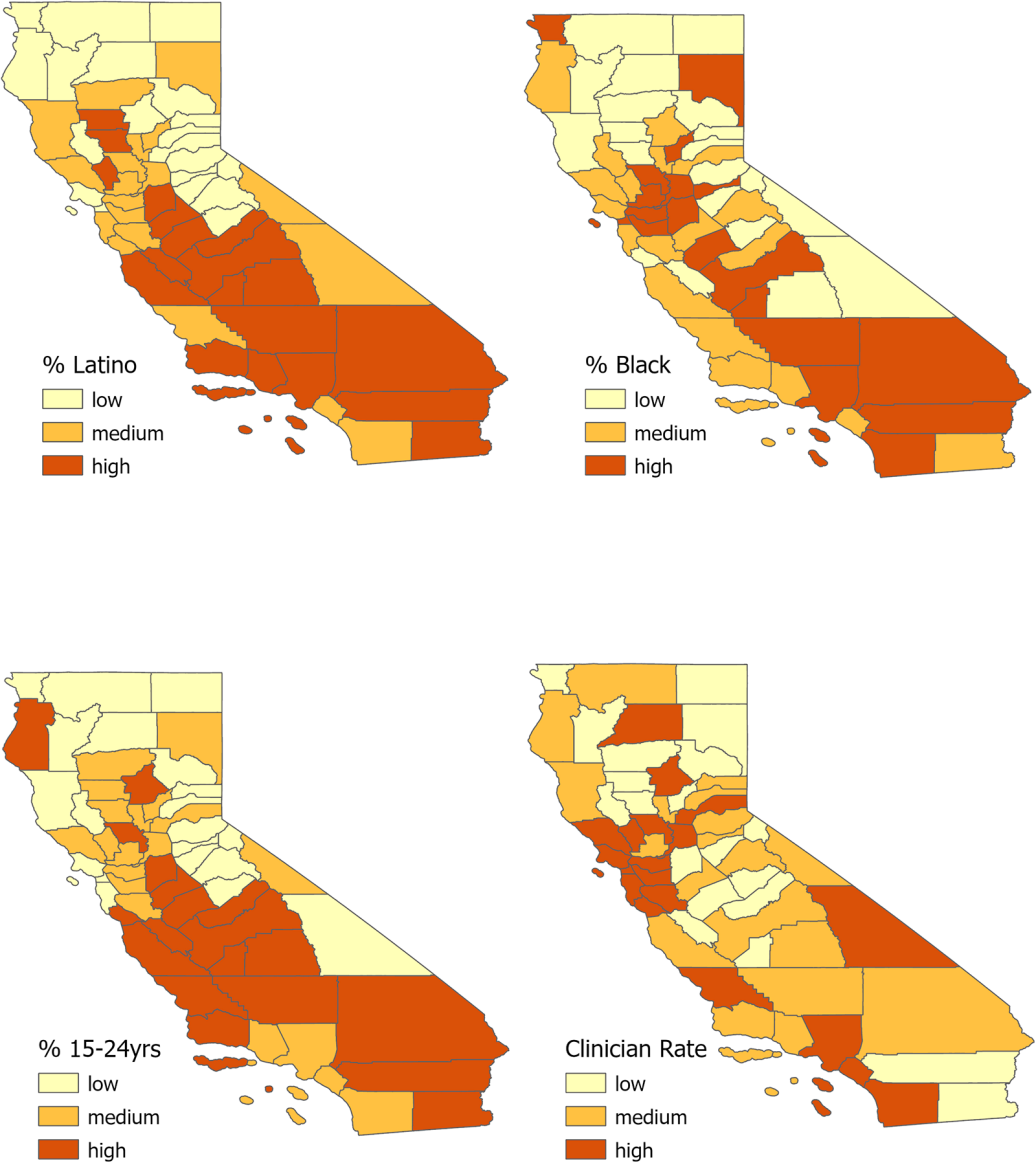
Tertile distribution (low, medium, high) of California county population by the following groups: (**A**) Latino individuals, (**B**) Black/African American individuals, (**C**) younger individuals (age 15–24 years), and (**D**). rate of health care providers per 100,000 individuals.

As shown in Figure 1, similar spatial patterns emerged in the demographic composition of California Counties. Counties in Central and Southern California had a high percentage (within the upper tercile) of Latino, Black/African American, and young people aged 15–24 years but a lower rate of healthcare providers with prescribing authority for HIV and STI home test kits.

**Figure 2:**
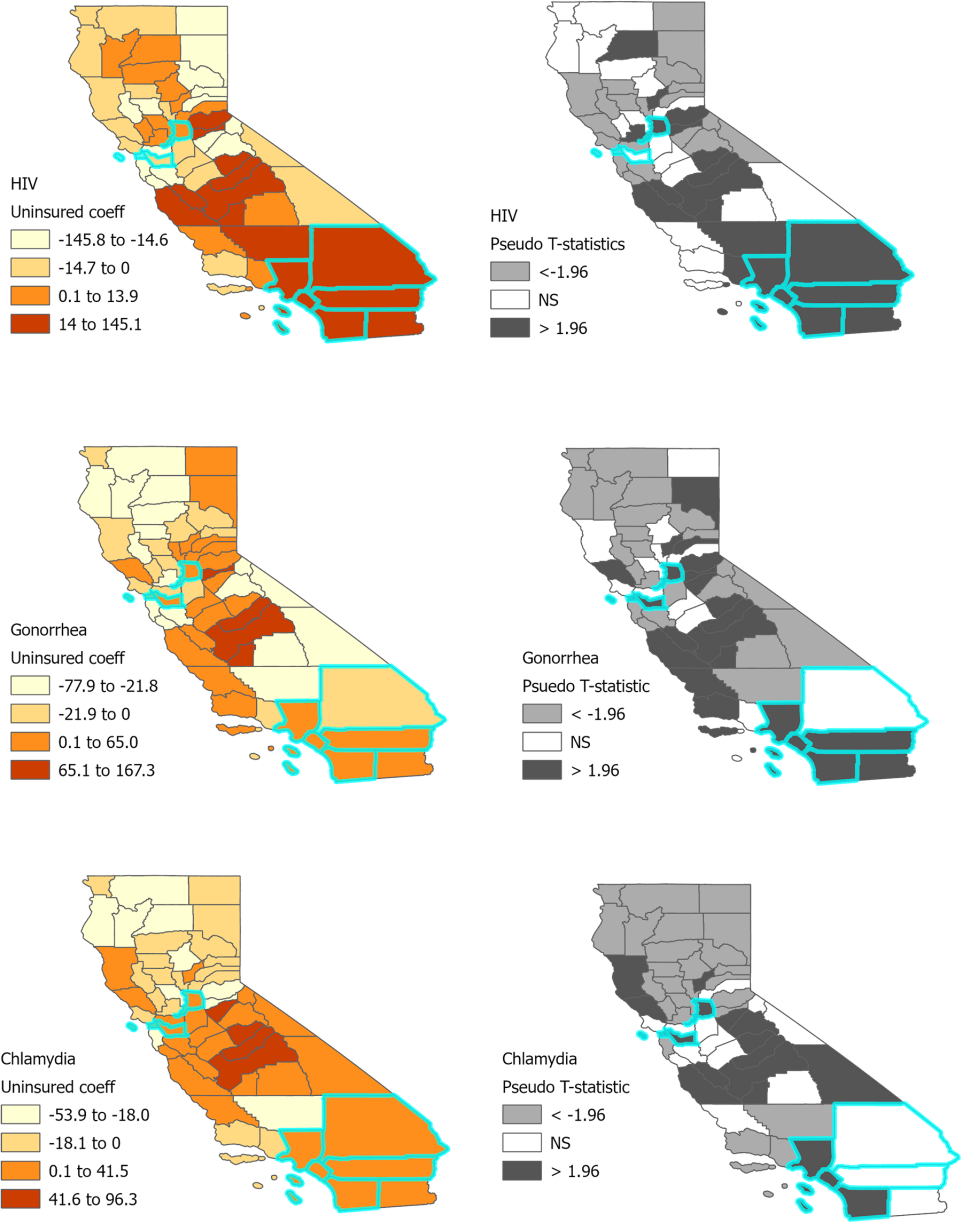
Bivariate associations between HIV/STIs and uninsured population in California counties. Areas outlined in turquoise are EHE jurisdictions.

Figure 2 displays the results of three separate GWPR models for each of the outcome variables. The left panel of Figure 2 shows the local regression coefficients describing the magnitude of the association between the percent uninsured and each outcome variable at the county level. Overlapping patterns emerge in that the percent uninsured is positively associated with HIV, gonorrhea, and chlamydia prevalence rates in Central and Southern California counties, the same counties that also had a higher proportion of Latino, Black/African American, and young adults. The right panel of Figure 2 displays the pseudo t-statistics where a *t* < −1.96 or *t* > 1.96 indicate a statistically significant association at a two-sided significance level of 0.05. The positive associations between the outcome variables and percent uninsured are significant in several of the Central and Southern California counties. In addition, the percent uninsured was significantly and positively associated with HIV, gonorrhea, and chlamydia prevalence rates in several EHE jurisdictions as indicated by the highlighted counties in Figure 2.

Table 2 displays the HIV and STI prevalence rates and percent uninsured across the EHE jurisdictions. All eight counties had HIV/STI prevalence rates that exceeded the state average. Further, all the EHE jurisdictions in Southern California (Los Angeles, Orange, Riverside, San Bernardino, and San Diego counties) had a higher percentage of uninsured residents compared to the state average. Both Riverside and San Bernardino counties had lower rates of healthcare providers compared to the state average.

**Table 2:** HIV prevalence rate, percent uninsured, and healthcare providers rate by EHE jurisdictions.

EHE jurisdiction	HIV cases per 100,000	Gonorrhea cases per 100,000	Chlamydia cases per 100,000	% Uninsured	Healthcare providers per 100,000
Alameda county	425	206	429	4.3	486.1
Los Angeles county	595	258	528	9.2	464.4
Orange county	264	143	342	7.1	487.1
Riverside county	474	160	442	8.5	233.2
Sacramento county	348	282	456	5.5	489.5
San Bernardino county	272	196	480	8.3	342.0
San Diego county	473	183	551	7.6	528.6
San Francisco county	1,515	473	659	3.6	1,154.3

## Discussion

Our ecologic study found that HIV, gonorrhea, and chlamydia rates were significantly and positively associated with the proportion of uninsured residents in several Central and Southern California counties. Several counties in Central and Southern California also had a high proportion of demographic groups who are highly vulnerable to HIV and other STIs. This includes a large proportion of Latino, Black/African American, and young residents between the ages of 15–24 years. However, most counties in Central and Southern California had a low density of healthcare providers with prescriptive authority to make home test kits available to these vulnerable communities, a requirement under California's new legislation. The shortage of healthcare providers in these counties seriously limits the potential reach and impact this legislation can have on remotely providing sexual health care to all Californians.

Senate Bill 306's requirement of an in-network clinician's prescription or standing order for home test kits to be covered by a health insurance plan appears to be a fatal flaw within the law. Nearly 1 in 15 Californians are uninsured, and approximately 5.3 million Californians live in a healthcare desert or do not have a usual source of healthcare (23, 27). An analysis of Senate Bill 306 by the California Health Review Benefits Program found that many health plans and insurers do not have systems in place to order or reimburse for home test kits (28). For example, billing codes have not been established to allow California's Medi-Cal program to begin paying for home testing kits. As of now, nearly 6.3 million Californians aged 12–64 years enrolled in Medi-Cal will not have access to home testing (29). Additionally, litigation at the federal level also threatens access to preventative services that are now available through the Affordable Care Act (ACA), the federal program that finances Medi-Cal. A recent decision (Braidwood v. Becerra) (30) from a U.S. District Court judge struck down provisions in the ACA that requires most health care plans to cover a range of preventive services without cost-sharing (deductibles or copays) for their enrollees (31, 32). Some of the preventive services affected include screening for hepatitis C and HIV. This recent court ruling at the national level could further jeopardize the rollout and potential impact of Senate Bill 306 within California.

The reality is that, rather than reduce barriers, Senate Bill 306 will more likely broaden disparities by perpetuating testing roadblocks for Californians without insurance coverage or access to primary health care. This means that the very groups who are at highest risk of HIV and STIs will be the ones least likely to access home testing under the new law. Our finding that HIV/STI rates increase in regions where the proportion of uninsured residents is higher is important given these associations were statistically significant in California's Central Valley, an area composed of agricultural counties that are largely rural, remote, with a high immigrant population (33, 34). Similar findings were identified in two landlock counties of Southern California, Riverside and San Bernardino counties, which have large rural pockets and are also EHE jurisdictions with substantially higher rates of HIV transmission compared to the national rates. Workforce shortages and limited resources in these rural areas create healthcare deserts which in turn compound the inequities experienced in California. Moreover, the redeployment of the STI workforce and testing supplies toward COVID-19 pandemic response efforts likely exacerbated California's HIV and STI epidemic (35). Indeed, the STI burden is not equal across the state of California: infection rates are greatest among racial and ethnic minorities, youth, and gay and bisexual men (2, 36). These groups and other marginalized Californians with low healthcare access will undoubtedly be left behind by Senate Bill 306, widening health disparities.

Other legislative efforts that attempt to lessen the rising tide of HIV infections in California have also been met with implementation barriers. In 2019, California's Senate Bill 159 provided pharmacists with the authority to furnish HIV prevention medications that include post-exposure prophylaxis (PEP) and a 60-day provision of pre-exposure prophylaxis (PrEP) to individuals without a prescription from a healthcare provider. With the removal of the prescribing requirement for PEP and PrEP by a healthcare provider, pharmacy delivery of HIV prevention medications has the potential of making these drugs available to the communities who could benefit. However, despite this change in policy, implementation of the law and associated regulations have been slow. A recent survey of pharmacists in California highlighted that after 4 years of the law being enacted, only 11% of pharmacist prescribed PrEP and 13% of pharmacist furnished PEP as authorized under Senate Bill 159 (37).

For the implementation of Senate Bill 306 to reach the communities that are disproportionately impacted by HIV and other STIs, the California legislature will need to remove the requirement that home test kits must be deemed medically necessary and prescribed by an in-network clinician or furnished through a standing order for patient use. Other programs have successfully implemented STI home testing kits, which do not require a clinician's prescription and allows for an individual to determine its medical necessity (38, 39). There is already such a program in California (Don't Think—Know) (40). However, it is limited to cis-gender women, age 12–24 years who reside in select California counties. Expanding programs that offer free home-based test kits, without a prescription requirement, to all Californians would address the key limitations of Senate Bill 306 and ensure that access to home testing is equitable and effective in ending the HIV and STI epidemic in the state.

There are some limitations to our study. First, all associations described are based on aggregate data at the county level and are subject to interpretation limitations due to the ecological fallacy. Our results are not evidence of an association or causal relationship capturing disparities in access to home test kits at the individual level. Further, HIV/STI prevalence rates, demographic composition, and healthcare resources can vary substantially across localities within a county. A more granular analysis would involve smaller spatial units to better understand these spatial relationships. Second, our spatial regression model was limited to one explanatory variable, proportion uninsured at the county-level. Due to multicollinearity issues between explanatory variables that is inherent with small sample sizes (*n* = 58 counties), we could not control for other demographic variables which could further explain the spatial variation of HIV/STI prevalence rates. However, using choropleth maps, we were able to characterize the demographic composition and healthcare environment of counties with a positive association between HIV/STI prevalent rates and proportion uninsured providing a more comprehensive understanding of which groups would be impacted by California's legislation.

In conclusion, cutting-edge solutions are needed to stem the rising tide of new STI and HIV infections in California. The provisions in Senate Bill 306 are well-intentioned, but its limitations will only make it a hindrance to meaningfully improving access to STI and HIV testing. The law will ensure that Californians living in coastal areas of the state with more access to healthcare providers will have access to home test kits while disenfranchising Black/African American, Latino, and younger Californians who live in the Central and Southern parts of the state facing an acute shortage of healthcare providers. While the state legislatures’ intentions with Senate Bill 306 was novel and innovative, the law as written still leaves many Californians behind. Our study highlights the need to invest in developing the healthcare workforce for California's Central Valley and Southern counties to ensure comprehensive sexual health care is provide to all under the new law.

## Data Availability

Publicly available datasets were analyzed in this study. This data can be found here: https://data.chhs.ca.gov/dataset/stds-in-california-by-disease-county-year-and-sex; https://aidsvu.org/; https://data.census.gov/; https://data.hrsa.gov/topics/health-workforce/ahrf.
